# Integrated analysis of potential biomarkers associated with diabetic periodontitis development based on bioinformatics: An observational study

**DOI:** 10.1097/MD.0000000000036019

**Published:** 2023-11-17

**Authors:** Yiran Wu, Yong-Hu Xing, Shuai Tao, Min Jiao, Min Zhu, Ya-Ting Han, Wei Guo, Xiu-Bin Tao

**Affiliations:** a Department of Nursing, The First Affiliated Hospital of Wannan Medical College, Wuhu, China; b Oral Medical Center, The First Affiliated Hospital of Wannan Medical College, Wuhu, China; c School of Basic Medicine, Wannan Medical College, Wuhu, China.

**Keywords:** bidirectional relationship, bioinformatics, diabetes, inflammation, periodontitis

## Abstract

Based on the importance of chronic inflammation in the pathogenesis of periodontitis and diabetes, the bidirectional relationship between these 2 diseases has been widely confirmed. However, the molecular mechanisms of bidirectional relationship still need to be studied further. In this study, gene expression profile data for diabetes and periodontitis were obtained from Gene Expression Omnibus (GEO) database. Integrative analytical platform were constructed, including common differentially expressed genes (cDEGs), Gene Ontology-Kyoto Encyclopedia of Genes and Genomes (GO-KEGG), and protein–protein interaction. Hub genes and essential modules were detected via Cytoscape. Key hub genes and signaling pathway that mediate chronic inflammation were validated by qPCR and Western blot. Eleven cDEGs were identified. Function analysis showed that cDEGs plays an important role in inflammatory response, cytokine receptor binding, TNF signaling pathway. As hub genes, *CXCR4, IL1B, IL6, CXCL2*, and *MMP9* were detected based on the protein–protein interactions network. *IL1B, CXCR4* mRNA were up-regulated in gingivitis samples compared with normal tissues (*P* < .05). Western blot indicated that the levels of TNF were enhanced in gingivitis of type 2 diabetes compared with normal tissues (*P* < .01). Hub gene and TNF signaling pathway are helpful to elucidate the molecular mechanism of the bidirectional relationship between periodontitis and diabetes.

## 1. Introduction

Periodontitis is an oral disease with a high incidence rate of about 11% globally and up to 50% population over 30 years old in the U.S., being the sixth most common human disease.^[1–3]^ The pathogenesis of periodontitis is complicated, and it is mainly a chronic inflammatory of periodontal tissue induced by bacteria.^[4]^ Its main features include loss of periodontal tissue support, manifested as clinical attachment loss and radiographically assessed alveolar bone loss, periodontal pocket and gingival bleeding.^[5]^ At present, periodontitis is difficult to treat and has high recurrence rate and morbidity, which has become an important factor threatening public health, and finding new potential therapeutic targets has become particularly urgent.

Coincidentally, the global prevalence of diabetes is about 8.5%, which is also a major public health challenge.^[6]^ Metabolic disorders in the course of diabetes can lead to a series of complications, including diabetic nephropathy, microangiopathy, and chronic inflammation.^[7–9]^ As research continues, a bidirectional relationship between diabetes and periodontitis has been established, namely that diabetes increases the risk of periodontitis, which has a negative impact on glycemic control.^[10]^ Studies have shown that diabetes is a risk factor for periodontitis and increases the severity;^[11]^ the prevalence of periodontitis in the diabetic population was 60%, significantly higher than that in the general population.^[12]^ Furthermore, controlling periodontal inflammation has the potential to reduce the systemic inflammation responsible for complications of diabetes.^[13]^ At present, research on the synergistic effect between periodontitis and diabetes is very limited. Studies have shown that RAC2, PTPRC, and FcγR-mediated phagocytic pathway have been implicated as a key component of in both periodontitis and diabetes pathogenesis.^[14]^ However, considering the complex interrelationship between periodontitis and diabetes, more molecular basis needs to be elucidated.

This study attempts to find the relationship and biological pathways between diabetes and periodontitis through bioinformatics. Related data sets were selected from GEO database and screened out common differentially expressed genes (cDEGs). Based on the cDEGs, further analysis was accomplished including gene ontology-Kyoto encyclopedia of gene and genome (GO-KEGG), protein–protein interactions (PPIs), and hub genes. Finally, key hub genes and signaling pathway were validated using clinical samples. Through the above studies, we explored the potential molecular mechanisms of the bidirectional relationship between diabetes and periodontitis, and providing new insights into the treatment and prevention of these 2 diseases.

## 2. Materials and methods

### 2.1. Clinical samples and ethics

Five cases of gingiva from diabetic periodontitis patients and 4 cases of normal gingiva were collected in the First Affiliated Hospital of Wannan Medical College. The inclusion criteria: excluding other systemic diseases except diabetes, only hypoglycemic treatment was accepted before surgery; bleeding on probing (+); probing pocket depth > 4mm; clinical attachment loss > 3mm. This study was approved by the Ethics Committee of Wannan Medical College (No. YJSHLB20230626). All methods were performed in accordance with the ethical standards of the Ethics Committee of Wannan Medical College and with the Helsinki declaration.

### 2.2. Retrieval of datasets

Four datasets on periodontitis (GSE10334, GSE16134, GSE24897, and GSE137351) and 2 datasets on type 2 diabetes (GSE9006 and GSE96804) were involved in this study, and all 6 datasets were from GEO database (https://www.ncbi.nlm.nih.gov/geo/). In GSE10334, the gene expression characteristics of 64 healthy gingival and 90 periodontitis gingival were analyzed.^[15]^ GSE16134 compared the correlation between subgingival bacterial profiles and gene expression in gingival tissues between 241 samples from 120 patients undergoing periodontal surgery and 69 healthy controls.^[16]^ *Porphyromonas gingivalis* is a keystone bacterial pathogen of chronic periodontitis;^[17]^ GSE14897 compared the gene expression profiles between macrophages exposed to *P gingivalis* and controls, and discussed the significance of this bacterium in acute and chronic periodontitis. GSE13735 analyzed the gene expression profile of neutrophils exposed to *filifactor alocis*.^[18]^ Gene expression changes in peripheral blood mononuclear cells from 12 patients with type 2 diabetes mellitus and 24 healthy controls were analyzed by GSE9006.^[19]^ GSE96804 was used to analyze the gene expression profiles of glomeruli from 41 patients with type 2 diabetes mellitus and 20 healthy people.^[20]^

### 2.3. Screening of cDEGs

**Figure 1.**
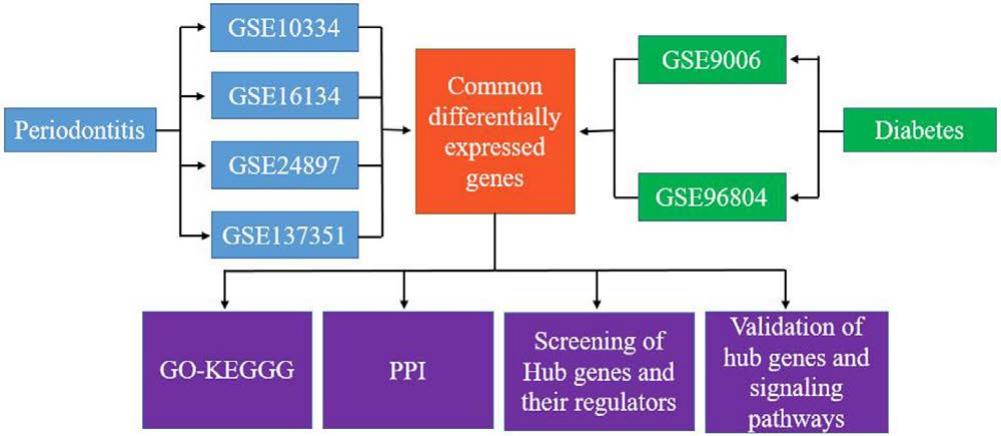
Study flow chart.

Differentially expressed genes for 6 datasets were analyzed through GEO2Rweb tool (https://www.ncbi.nlm.nih.gov/geo/geo2r/) and limma package. cutoff criteria was obtained by adjusted *P* value < .05 and log2FC > 0.75 or < −0.75. Genes unique and common to each dataset were analyzed, and the results were visualized using ggplot2 [3.3.6] and VennDiagram [1.7.3]. The flow chart of this study is shown in Figure 1.

### 2.4. GO-KEGG enrichment analysis of cDEGs

The functional annotation of GO, including biological process (BP) and molecular function (MF), was performed by the open access WebGestalt tool, as well as the KEGG pathway enrichment analysis (http://www.webgestalt.org). False discovery rate ≤ 0.05 were considered statistically significant. ClusterProfiler package (v.3.14.3) was used for enrichment analysis while Org.Hs.e.g..db package (v.3.10.0) was for ID conversion.

### 2.5. Network establishment of cDEGs

Genes with higher expression differences are considered to be key genes that mediate disease progression. So, PPI of encoding by cDEGs was analyzed by STRING (v11.5, https://www.string-db.org/). Parameter setting: organism selects *Homo sapiens*, required score selects medium confidence (0.400), false discovery rate stringency chooses medium (5%).

### 2.6. Identification of hub genes and module analysis

PPIs were analyzed via Cytoscape software, and the hub genes were calculated via 6 topological algorithms. The nodes that have the most interactions were considered to be a hub gene. Molecular Complex Detection plugin of Cytoscape software was used to detect the most profound modules from the PPIs network.

### 2.7. Screening of hub genes biological regulators

NetworkAnalyst database (https://www.networkanalyst.ca/) was used to identify transcription factor (TF)-miRNA co-regulatory network. The common network topology measures were also computed based on well-established igraph R package. TF and gene target data derived from the ENCODE ChIP-seq data. Only peak intensity signal < 500 and the predicted regulatory potential score < 1 were taken into account. Drugs targeting hub genes were screened by over representation analysis method in Drugbank of WebGestalt database (http://webgestalt.org/).

### 2.8. Quantitative PCR (qPCR) for inflammation and normal tissues

Five gingivitis samples of type 2 diabetes patients and 4 normal gingiva samples were collected from The First Affiliated Hospital of Wannan Medical College. Refer to relevant literature,^[21]^ the qPCR was performed and calculated by means of 2−ΔΔCt methods. The main reagents and instruments are as follows: TRIzol reagent (Tiangen, U8428), HiScript II Q RT SuperMix (Vazyme Biotech, R222-01), ChamQ universal SYBR qPCR Master Mix (Vazyme Biotech, Q711-02), Real-Time PCR Detection System (Roche, LightCycler 96). Related primers were displayed as following: IL1B, F: 5′-CCACAGACCTTCCAGGAGAATG-3′, R: GTGCAGTTCAGTGATCGTACAGG; CXCR4, F: 5′-CTTCTTAACTGGCATTGTGG-3′, R: 5′-GTGATGACAAAGAGGAGGTC-3′, GAPDH, F: 5′-GAAGGTGAAGGTCGGAGTC-3′, R: 5′-GAAGATGGTGATGGGATTTCC-3′. The comparison of expression levels of inflammation and normal tissues were analyzed by unpaired *t* test. *P* < .05 was considered statistically significant.

### 2.9. Western blot (WB) analysis

Total proteins of gingivitis samples were extracted and protein concentration was detected, WB assay was performed. The primary antibodies used were as follows: TNF antibody (proteintech, 21574-1-AP) (diluted at 1:5000), GAPDH (proteintech, 80570-1-RR) (diluted at 1:5000), HRP-labeled goat antirabbit IgG (Beyotime, A0208) (diluted at 1:2000). The signal was detected by chemiluminescence, and the bands’ gray value was measured by ImageJ software.

## 3. Results

### 3.1. Identification of cDEGs between periodontitis and diabetic

**Figure 2.**
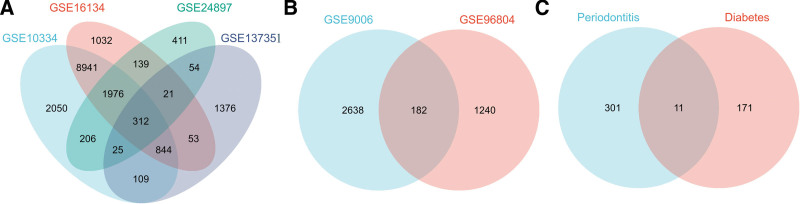
Venn diagram of common differentially expressed genes identified in individual cohorts.

As regards the periodontitis, a total of 312 cDEGs were identified from the 4 datasets (Fig. 2A). For diabetes, a total of 182 cDEGs were identified from the 2 datasets (Fig. 2B). Further analysis showed that a total of 11 cDEGs were identified from the above 6 datasets, including ARMCX6, CXCL2, CXCR4, DOCK4, IL1A/1B/6, KMO, MMP9, SERTAD2, and VEGFA (Fig. 2C). The detailed expression characteristics of these 11 cDEGs in each dataset are shown in Table S1, Supplemental Digital Content, http://links.lww.com/MD/K756.

### 3.2. GO-KEGG enrichment analysis of cDEGs

The GO analysis was conducted for enrichment of 11 cDEGs’ function and pathway. In terms of BP, these cDEGs were mainly involved in leukocyte migration and cell chemotaxis. As for MFs, cDEGs were involved in cytokine activity. KEGG enrichment analysis on the 11 cDEGs showed that the top 3 signal pathways which were significantly enriched were in Rheumatoid arthritis, IL-17, and AGE-RAGE signaling pathway. Detailed GO-KEGG enrichment information of cDEGs was summarized in Table 1.

**Table 1:** GO-KEGG enrichment analysis of common differentially expressed genes.

Ontology	ID	Description	GeneRatio	*P*. adjust
BP	GO:0050900	Leukocyte migration	7/10	1.95e−07
BP	GO:0060326	Cell chemotaxis	6/10	2.56e−06
BP	GO:0071222	Cellular response to lipopolysaccharide	5/10	1.46e−05
BP	GO:0071219	Cellular response to molecule of bacterial origin	5/10	1.46e−05
BP	GO:0097529	Myeloid leukocyte migration	5/10	1.46e−05
CC	GO:0005741	Mitochondrial outer membrane	2/11	.0606
CC	GO:0031968	Organelle outer membrane	2/11	.0606
CC	GO:0019867	Outer membrane	2/11	.0606
MF	GO:0005125	Cytokine activity	5/10	5.13e−06
MF	GO:0005126	Cytokine receptor binding	5/10	5.31e−06
MF	GO:0070851	Growth factor receptor binding	4/10	1.39e−05
MF	GO:0048018	Receptor ligand activity	5/10	4.14e−05
MF	GO:0030546	Signaling receptor activator activity	5/10	4.14e−05
KEGG	hsa05323	Rheumatoid arthritis	5/9	1.82e−06
KEGG	hsa04657	IL-17 signaling pathway	4/9	7.39e−05
KEGG	hsa04933	AGE-RAGE signaling pathway in diabetic complications	4/9	7.39e−05
KEGG	hsa04668	TNF signaling pathway	4/9	8.73e−05
KEGG	hsa04060	Cytokine-cytokine receptor interaction	5/9	.0001

BP = biological process; CC = cellular component; MF = molecular function; KEGG = Kyoto Encyclopedia of Genes and Genomes.

### 3.3. Construction of PPI network

**Figure 3.**
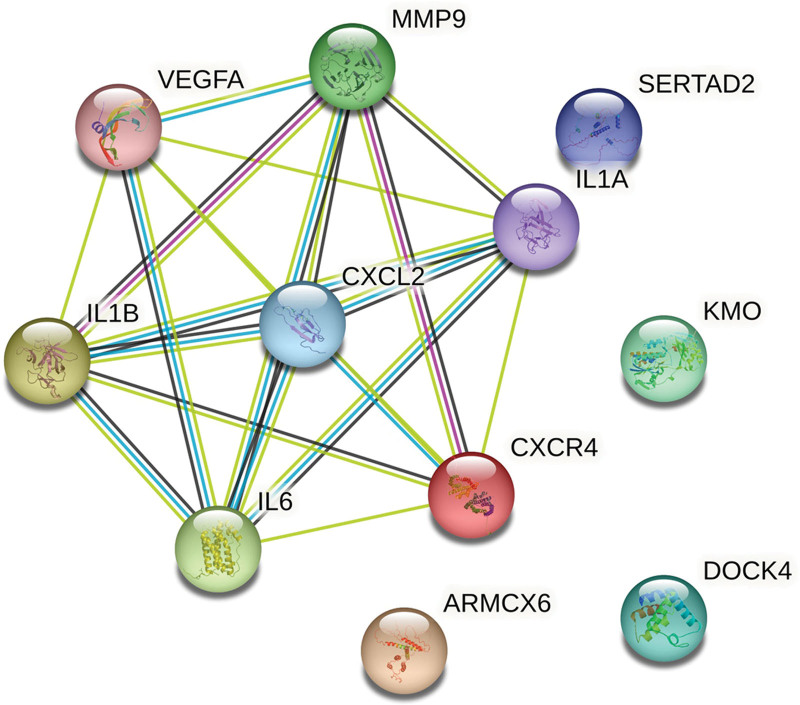
PPI network of cDEGs. Nodes represent proteins, colored nodes: query proteins and first shell of interactors. Edges represent protein–protein associations, purple and green represent known interactions that have been verified. cDEGs = common differentially expressed genes, PPI = protein–protein interaction.

A total of 11 nodes and 21 edges were mapped in the PPI network of the identified cDEGs. PPI enrichment *P*-value: 9.63e−7; average node degree: 3.82, avg. local clustering coefficient: 0.636. As shown in Figure 3, the cDEGs showed significant interactions, such as IL1B, IL6, CXCR4 etc.

### 3.4. Identification of hub genes and module analysis

**Figure 4.**
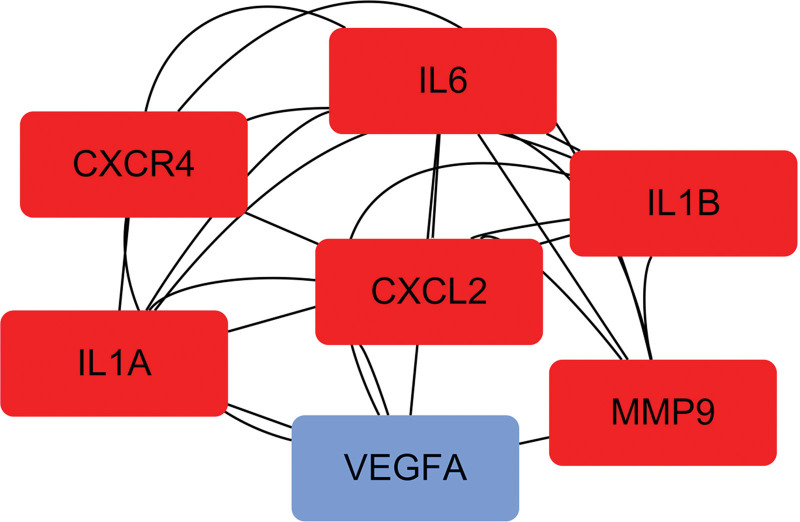
Module analysis network obtained from PPI. The network represents highly interconnected regions of the PPI. CXCR4, IL1A/1B/6, CXCL2, and MMP9 are highlighted in red color, which are both hub genes and key modules. The interaction frequency of blue (VEGFA) in modules is lower than red. PPI = protein–protein interaction.

It was analyzed by different topological calculation methods in CytoHubba, CXCR4, VEGFA, CXCL2, IL1A, IL1B, IL6, and MMP9 were considered as hub genes due to their high frequency and scores (Table 2). Module analysis showed that CXCR4, IL1A/1B/6, CXCL2, and MMP9 were high-density modules, including 7 spots and 28 edges (Fig. 4).

**Table 2:** Hub genes were obtained by 6 topology calculation methods.

MMC	DMNC	MNC	Degree	EPC	EcCentricity
CXCR4	CXCR4	CXCR4	CXCR4	IL6	CXCR4
VEGFA	VEGFA	VEGFA	VEGFA	MMP9	VEGFA
CXCL2	CXCL2	CXCL2	CXCL2	IL1B	CXCL2
IL1A	IL1A	IL1A	IL1A	CXCR4	IL1A
IL1B	IL1B	IL1B	IL1B	VEGFA	IL1B
MMP9	MMP9	MMP9	MMP9	IL1B	MMP9
IL6	IL6	IL6	IL6	CXCL2	IL6

MMC, DMNC, MNC, etc represents 6 topology calculation methods. The score of hub genes gradually decreased from top to bottom.

### 3.5. Biological regulators of hub genes

**Figure 5.**
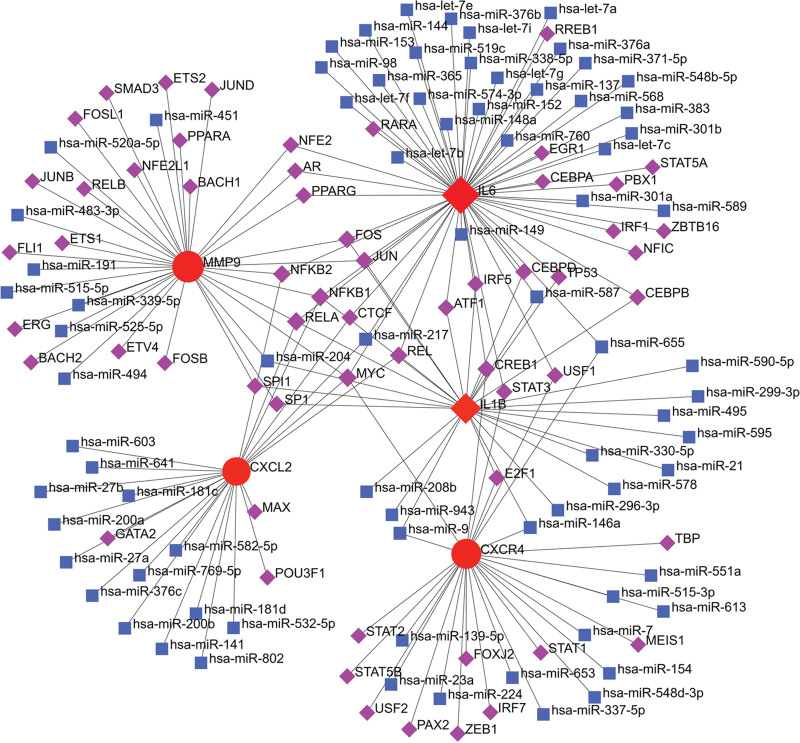
TF-miRNA co-regulatory network. The network consists of 141 nodes and 175 edges. The nodes in red color are the hub genes, the blue nodes represent miRNA, the green nodes represent TFs. TF = transcription factor.

**Figure 6.**
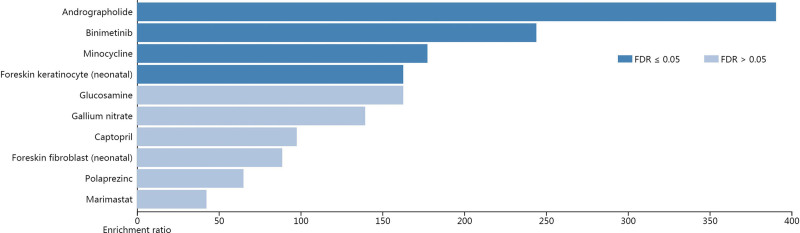
Suggested top drug compounds for hub genes via WebGestalt database.

By NetworkAnalyst analysis, 307 TFs regulating 5 hub genes expression were identified. The main TFs regulating hub genes include MYC, RELA, GATA2, NFKB1-2, CREB1, etc. Moreover, 1414 miRNAs that regulated the expression level of hub genes were identified, among which frequency of co-regulation of hsa-miR-655, hsa-miR-217, hsa-miR-146a, hsa-miR-9, and hsa-miR-204 with TFs were higher (Fig. 5). According to WebGestalt database analysis, andrographolide, binimetinib, and minocycline target hub genes (Fig. 6).

### 3.6. Validation of key hub genes and TNF signaling pathways

**Figure 7.**
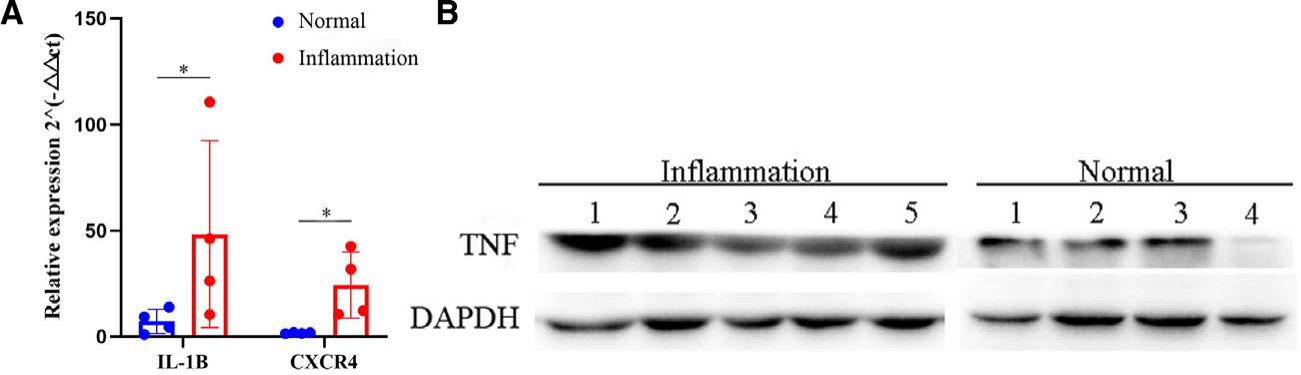
Validation of key genes and signaling pathways by qPCR and WB. (A) qRT-PCR results of IL1B and CXCR4 mRNA expression in gingivitis samples (n = 5) and normal gingiva samples (n = 4); *P < .05. (B) Expression levels of TNF in gingivitis samples (n = 5) and normal gingiva samples (n = 4) were detected by WB. The samples derive from the same experiment and that gels/blots were processed in parallel. The picture has been cropped and the original blots/gels are presented in Figures S1, Supplemental Digital Content, http://links.lww.com/MD/K757, S2, Supplemental Digital Content, http://links.lww.com/MD/K758, S3, Supplemental Digital Content, http://links.lww.com/MD/K759, S4, Supplemental Digital Content, http://links.lww.com/MD/K760. WB = western blot.

We utilized qPCR to validate the hub genes IL1B, CXCR4 mRNA expression and found the IL1B, CXCR4 mRNA were up-regulated in gingivitis samples compared with normal tissues (Fig. 7A, *P* < .05). To determine the effects on the TNF signaling pathway in the gingivitis of type 2 diabetes, the levels of TNF was measured by WB. The bands gray value of inflammation group 1 to 5 significantly higher than the normal group 1 to 4 (*P* < .01), and indicate that the levels of TNF were enhanced in gingivitis of type 2 diabetes compared with normal tissues (Fig. 7B).

## 4. Discussion

Based on the importance of chronic inflammation in the pathogenesis of periodontitis and diabetes, a bidirectional relationship between these 2 diseases has been widely confirmed; namely, diabetes increases the risk of periodontitis and the difficulty of treatment, and periodontitis has a negative impact on controlling blood glucose.^[10,22–24]^ This bidirectional relationship suggests that the pathogenesis of the 2 diseases overlap to some extent, but more molecular mechanisms have been rarely reported. In this study, bioinformatics methods were used to analyze the datasets related to the 2 diseases, and 11 cDEGs were identified. Coincidentally, the expression trends of cDEGs in the 2 diseases were almost the same (Table S1, Supplemental Digital Content, http://links.lww.com/MD/K756). In addition, GO-KEGG enrichment analysis showed that in BP and MF, cDEGs had functions such as leukocyte chemokine and chemokine binding; KEGG signaling pathway suggested that IL-17 signaling pathway and TNF signaling pathway were significantly enriched; these enriched are associated with inflammatory responses and provide a molecular basis for the bidirectional relationship between periodontitis and diabetes.

Further study of PPI network and module analysis of cDEGs revealed that these genes not only had high frequency interactions with each other, but also had high frequency interactions with other genes, which suggested that cDEGs played an important role in the pathogenesis process. Importantly, we discovered hub genes *CXCR4, IL6, IL1B, CXCL2, MMP9* through comprehensive topological analysis. Studies have shown that high expression of *CXCR4*/*CXCL12* axis can recruit M1 macrophages, produce inflammatory cytokines, and cause insulin resistance;^[25]^ meanwhile, this pathway mediates the absorption of alveolar bone during the pathogenesis of chronic periodontitis.^[26]^ Thamiris et al demonstrated that *IL6* and *IL1B* are important molecules for the coexistence of periodontitis and type 2 diabetes mellitus;^[27]^ high expression of *IL6* and *IL1B* increases susceptibility to diabetes and periodontitis.^[28,29]^ *MMP9* is an important biomarker to define the grade of periodontitis,^[30]^ overexpression of which can promote the generation of osteoclasts, increase the level of periodontal inflammatory factors, and participate in alveolar bone absorption and periodontal tissue destruction;^[31,32]^ continuous hyperglycemia can induce *MMP9* production.^[33]^ This may be an important reason that causes diabetic periodontitis to be difficult to cure and easy to relapse. Interestingly, *HGF, PTPRC*, etc. have been found to be the hub genes of diabetic periodontitis,^[14]^ there are differences with the results of this study. Analyze the possible reasons for this difference: ① different data sets were applied to the study; ② the topology calculation methods of hub genes screening are different; ③ the pathogenesis of diabetic periodontitis is complex and may involve more genes.

Hub genes are important genes that affect the expression of a large number of genes and the activity of transcription factors, and play a key role in the process of disease; highly correlated hub genes affect the entire PPI network and can be used as potential targets for disease treatment.^[34]^ In this study, clinical samples of 5 patients with diabetic periodontitis were selected for verification by qPCR, and the expression of hub genes was consistent with the results of bioinformatics analysis, which further demonstrated the reliability of results. In addition, KEGG enrichment analysis suggested that cDEGs were significantly enriched in TNF signaling pathway, and numerous studies have shown that TNF signaling pathway is an important pro-inflammatory pathway that mediates chronic inflammation.^[35–37]^ TNF activation of pro-inflammatory and tissue damage-related pathways is mainly mediated by TNFR1.^[38]^ The expression of TNF was verified via WB, and the results showed that the TNF was overexpressed in gingivitis samples. This result suggests that TNF signaling pathway not only mediates chronic inflammation in diabetic periodontitis, but also plays an important role in the bidirectional effect of the 2 diseases.

TFs, miRNA, and drugs are important factors regulating hub genes. In this study, it was found that TFs such as MYC, RELA, GATA2, and NFκB1-2 and miRNA such as miR217 and miR-146a have regulatory effects on hub genes, among which RELA and NFκB1-2 almost regulated all hub genes. Studies show that RELA and NFκB1 are involved in the regulation of differentially expressed genes in type 2 diabetes mellitus^[39]^; RELA drives the expression of pro-inflammatory markers in periodontitis macrophages, and is highly expressed in the gingiva of type 2 diabetic periodontitis patients;^[40]^ GATA2 mediates the pathogenesis of periodontitis by regulating the expression of endothelin-A receptor.^[41]^ miR217 can reduce the expression of pro-inflammatory factors TNF-α, IL6, and IL10 through TLR4/PI3K/Akt/NF-κB signaling pathway, and inhibited inflammatory responses^[42]^; miR-146a has been shown to be an important negative regulator of immune responses through targeting 2 key genes, that is, TNF Receptor-Associated Factor 6 (TRAF-6) and IL-1 Receptor-Associated Kinase 1 (IRAK-1)^[43]^; the above research supports the results of this study. In addition, andrographolide and minocycline were found to target hub genes; previous studies have shown that andrographolide improves pneumonia and fibrosis by inhibiting AIM2 inflammasome^[44]^; minocycline is widely used in the treatment of dermatitis, periodontitis, atherosclerosis, and autoimmune diseases due to its anti-inflammatory, antiapoptotic, and angiogenic activities.^[45]^ These studies further prove that our prediction results are logical and will play a positive predictive role for clinical treatment.

In summary, this study analyzed the data sets related to diabetes and periodontitis by bioinformatics, screened out the cDEGs of the 2 diseases, analyzed the function and signal pathway of these genes, and calculated the hub genes on this basis. Then, the regulatory factors targeting hub genes had also been predicted (including TFs, miRNA, and drugs). Finally, we verified the hub genes and signal pathway with clinical cases. Through the above study, we explored the molecular basis and pathogenesis of the bidirectional effect between diabetic and periodontitis, which aiming to provide new light on prevention and treatment of the disease.

## Author contributions

**Data curation:** Shuai Tao, Min Jiao, Min Zhu, Yating Han.

**Formal analysis:** Min Jiao, Min Zhu.

**Investigation:** Yonghu Xing, Wei Guo.

**Methodology:** Yiran Wu, Wei Guo, Xiubin Tao.

**Project administration:** Xiubin Tao.

**Resources:** Yonghu Xing.

**Software:** Shuai Tao.

**Supervision:** Xiubin Tao.

**Writing – original draft:** Yiran Wu..

**Writing – review & editing:** Xiubin Tao.

## Supplementary Material










