# Supplementary figures and images for: Integrated analysis of potential biomarkers associated with diabetic periodontitis development based on bioinformatics: An observational study
Source: Medicine (Baltimore). 2023 Nov 17;102(46):e36019. doi: 10.1097/MD.0000000000036019 (PMC10659692; doi:10.1097/MD.0000000000036019)

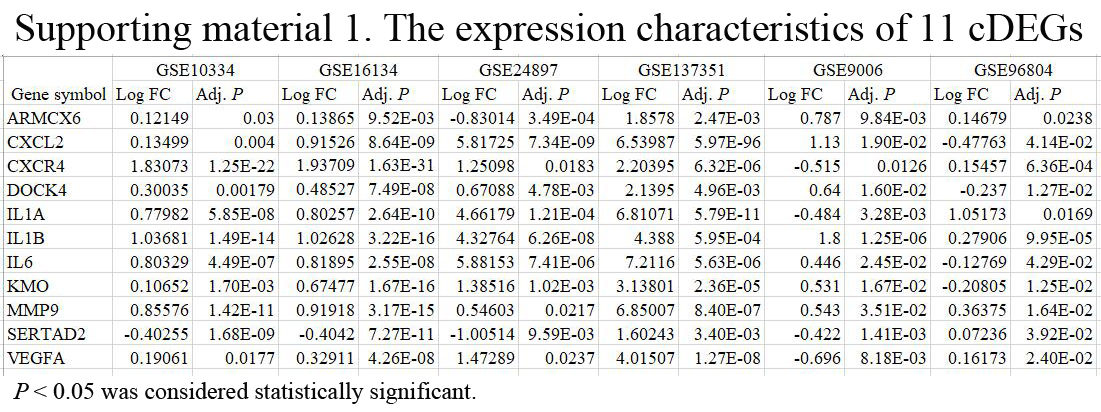

Supplement: Supplementary file 1 [file medi-102-e36019-s001.tif]

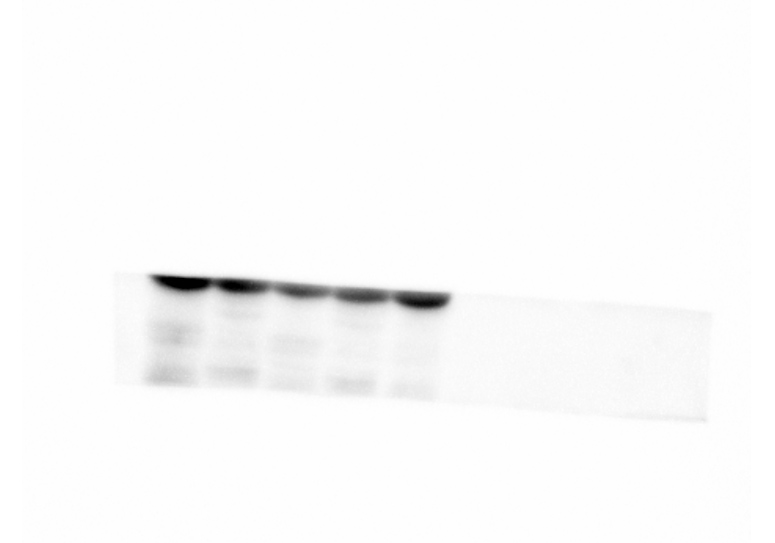

Supplement: Supplementary file 2 [file medi-102-e36019-s002.tif]

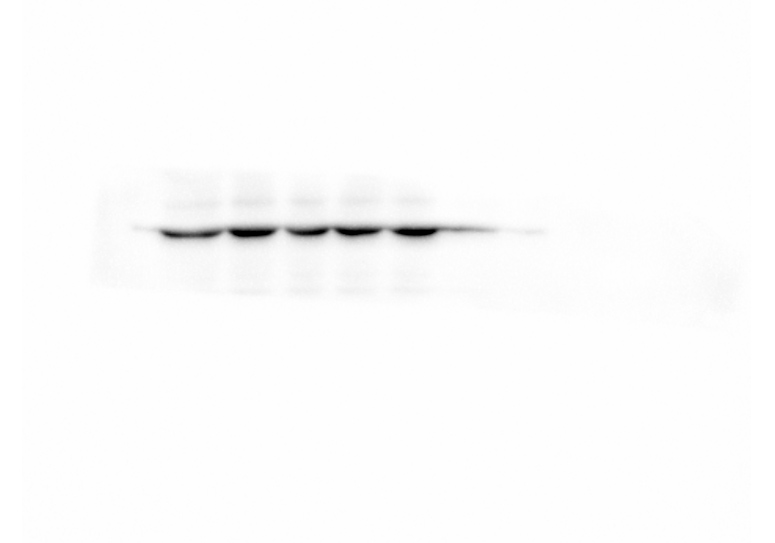

Supplement: Supplementary file 3 [file medi-102-e36019-s003.tif]

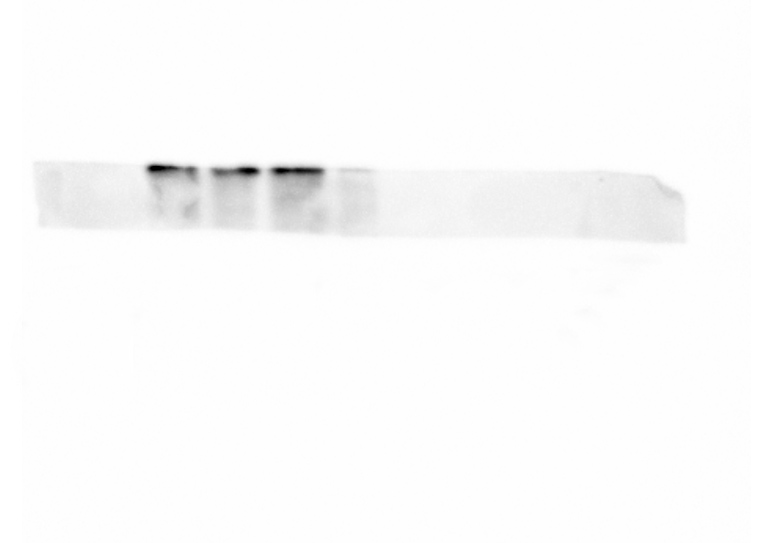

Supplement: Supplementary file 4 [file medi-102-e36019-s004.tif]

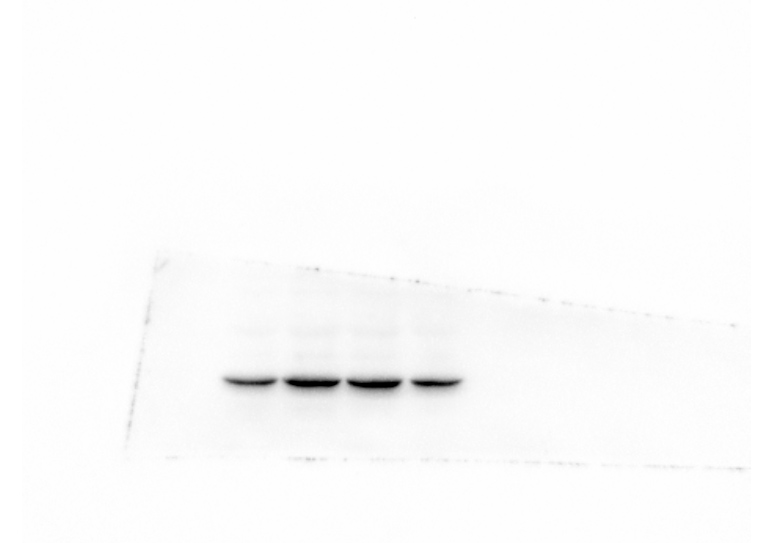

Supplement: Supplementary file 5 [file medi-102-e36019-s005.tif]
